# Embedded Transdermal Alcohol Detection via a Finger Using SnO_2_ Gas Sensors

**DOI:** 10.3390/s21206852

**Published:** 2021-10-15

**Authors:** Fatima Ezahra Annanouch, Virginie Martini, Tomas Fiorido, Bruno Lawson, Khalifa Aguir, Marc Bendahan

**Affiliations:** 1 Departament d’Enginyeria Electronica, Universitat Rovira i Virgili, Països Catalans 26, 43007 Tarragona, Spain; 2 Aix Marseille University, Université de Toulon, CNRS, IM2NP, UMR 7334, 13397 Marseille, France; virginie.martini@im2np.fr (V.M.); tomas.fiorido@im2np.fr (T.F.); bruno.lawson@im2np.fr (B.L.); khalifa.aguir@im2np.fr (K.A.)

**Keywords:** gas sensors, transdermal alcohol detection, blood alcohol contents, non-invasive detection, real time monitoring, SnO_2_

## Abstract

In this paper, we report the fabrication and characterization of a portable transdermal alcohol sensing device via a human finger, using tin dioxide (SnO_2_) chemoresistive gas sensors. Compared to conventional detectors, this non-invasive technique allowed us the continuous monitoring of alcohol with low cost and simple fabrication process. The sensing layers used in this work were fabricated by using the reactive radio frequency (RF) magnetron sputtering technique. Their structure and morphology were investigated by means of X-ray spectroscopy (XRD) and scanning electron microscopy (SEM), respectively. The results indicated that the annealing time has an important impact on the sensor sensitivity. Before performing the transdermal measurements, the sensors were exposed to a wide range of ethanol concentrations and the results displayed good responses with high sensitivity, stability, and a rapid detection time. Moreover, against high relative humidity (50% and 70%), the sensors remained resistant by showing a slight change in their gas sensing performances. A volunteer (an adult researcher from our volunteer group) drank 50 mL of tequila in order to realize the transdermal alcohol monitoring. Fifteen minutes later, the volunteer’s skin started to evacuate alcohol and the sensor resistance began to decline. Simultaneously, breath alcohol measurements were attained using a DRAGER 6820 certified breathalyzer. The results demonstrated a clear correlation between the alcohol concentration in the blood, breath, and via perspiration, which validated the embedded transdermal alcohol device reported in this work.

## 1. Introduction

Excessive alcohol consumption is a serious public health and safety issue worldwide. It can negatively affect human health across the lifespan by causing severe illnesses, namely: alcoholic hepatitis; liver cirrhosis; cancers (including throat, esophagus, liver, and breast); and high blood pressure [1,2,3]. Apart from this long list of health risks, it has great social and economic impacts [4,5]. Indeed, the consumers of alcohol do not only affect themselves but also affect those around them, for example by causing traffic accidents, violent behavior, inappropriate sexual behavior, and joblessness. Thus, it is essential to control alcohol consumption by a continuous monitoring of alcohol content in the human body.

Alcohol content can be detected immediately by examining blood, breath, urine, or saliva samples [6,7,8]. Blood testing is the most reliable method and is often considered as a reference for all control techniques. This invasive technique is accurate, reproducible, and reliable. However, it has several disadvantages, such as its cost; portability; the need for qualified personnel to collect and store the blood samples; the stages of the pre-treatment of the samples; and the extensive amount of time required to obtain the results (12 to 24 h). Similar disadvantages were faced by techniques based on the collection of saliva and urine samples [9,10].

Breath analysis is a non-invasive technique that indirectly deduces the blood alcohol concentration (BAC) through the exhaled air [11]. According to Henry’s law, this concentration can be estimated with a factor of 2100 compared to the breath alcohol concentration (BAC) [12]. The main advantages of this indirect measurement technique are the portability and rapid estimation of the blood alcohol level. Nevertheless, several drawbacks are to be noted: the influence of external parameters, such as the temperature of the upper airways which can oscillate at around 34 °C; body temperature; the expiration rate; and the non-continuous monitoring of the BAC [13,14]. To overcome these shortcomings, non-invasive transdermal detection has been launched as a new route for the indirect monitoring of blood alcohol content through the alcohol emitted from the skin [15,16,17]. It was reported that once the alcohol is ingested, the human body excretes 1% of the consumption via the sweat (human perspiration), 0.7% through the breath, 0.3% in the urine, and mostly over 98% is metabolized by the liver [18].

In 1985, an impressive study was conducted by the Indiana School of Medicine, in which a temperature of 37 °C was maintained in a polyethylene bag and placed around the hand of volunteers who consumed a certain amount of alcohol [19]. The collected gas samples were analyzed using gas chromatography and compared to the concentration of ethanol in the exhaled air and in the blood. This study concluded that ethanol vapor can be easily removed through the skin by insensitive perspiration, in a sufficient quantity, to allow a reliable estimation of blood alcohol levels. Few years ago, a new era of transdermal ethanol measurement devices was inaugurated. Two notable bracelets based on electrochemical sensors were fabricated: WrisTAS™ by Giner, Inc. and the Secure Continuous Remote Alcohol Monitor (SCRAM) by Alcohol Monitoring Systems (AMS). Several studies have devoted themselves to studying the performance of these bracelets [20,21,22]. The bracelets have shown that, on different volunteers, transdermal alcohol can be strongly correlated with the magnitude and rate of blood alcohol concentration. Moreover, they noticed a delay of 30 to 120 min between the two concentration peaks, which can be related to different parameters, such as the amount of alcohol consumed; human metabolism; sex; and the frequency of consumption [20]. Even though these devices made it possible to evaluate blood alcohol levels non-invasively and continuously, they were bulky; expensive; and based on electrochemical sensors that usually require regular recalibration, unlike metal oxide microsensors.

Tin dioxide (SnO_2_) is a transparent semiconducting oxide with a band gap of 3.6 eV. It is a native n-type semiconductor with attractive electrical, optical, and electrochemical properties that allow it to be widely used in many applications, such as catalysis; gas sensors; and light emitting devices. As a sensing film, it has demonstrated an excellent gas sensing performance (i.e., high sensitivity and good stability) to a wide spectrum of reducing and oxidizing gases, such as ethanol; acetone; H_2_; and NO_2_. Various techniques were used to deposit SnO_2_ material, for instance sol-gel, chemical vapor deposition (CVD), metal organic deposition, and radio frequency (RF) sputtering. The latter was adopted in this work due to its successful use in the processing of thin, adherent, stable, and reproducible films [23,24].

In this paper, we fabricated an embedded transdermal alcohol detection device, using MEMS tin dioxide gas microsensors. First, SnO_2_ thin films were deposited directly onto the MEMS-based sensing platforms using reactive radio frequency (RF) magnetron sputtering. Processing parameters, such as oxygen partial pressure and operating temperature, were optimized to fulfil the best detection performances, including high sensitivity and stability with a fast detection rate, and a good resistivity to moisture. Subsequently, lab-calibration measurements were conducted by exposing the fabricated sensors to different concentrations of ethanol in dry and humid atmospheres. Finally, real time transdermal alcohol measurements were obtained using a volunteer (an adult researcher from our research group), by drinking 50 mL of tequila while breath alcohol measurements were simultaneously collected using the DRAGER 6820 certified breathalyzer, in order to compare and validate the results.

## 2. Materials and Methods

### 2.1. MEMS-Based Microsensor Platforms

**Figure 1:**
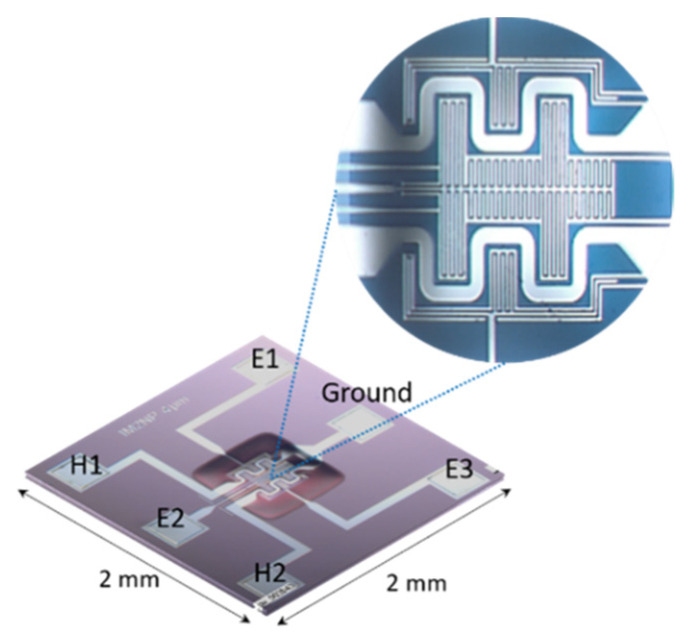
Photographs of the MEMS-based microsensor platform.

Figure 1 illustrates a MEMS-based microsensor platform fabricated in our laboratory under the patent FR 13 59494, 2013—US20160238548A1, 2016. It contains one membrane of 400 μm × 400 μm, in which interdigitated electrodes and two heaters in platinum were designed using clean room facilities and various microfabrication steps. The gap between the electrodes and their width is 4 μm, the resistance of each heater is 100 Ω, and the temperature coefficient is 3 × 10^−3^/K [25,26].

### 2.2. Material Deposition

50 nm of SnO_2_ sensing films was directly deposited over the MEMS substrates via reactive radio frequency (RF) magnetron sputtering. A metal target of 99.99% purity was used. The substrates were cleaned with acetone and then with ethanol, dried with air, and then placed inside the shadow mask. After a high vacuum, the pressure was fixed at 22 × 10^−3^ mbar by adjusting the Ar/O_2_ ratio. The substrate was covered and a pre-sputtering of about 1 h was started with the aim to homogenize the reactive plasma environment. At the deposition time, the cover was removed and the substrate holder was positioned in front of the target for a given time, depending on the desired thickness. After the deposition, the substrates were annealed to obtain stable nanostructured thin films. It is worth noting that the substrate temperature was maintained at room temperature during the whole process of deposition and the RF sputtering power was fixed at 200 W. The thickness of the deposited film was measured using a DEKTAK 6 M mechanical profilometer.

### 2.3. Material Characterization Techniques

The XRD analysis of the deposited SnO_2_ layers was carried out using a PAN analytical X’Pert Pro MPD diffractometer with a copper Kα line wavelength of λcu = 0.15418 nm. The spectra were analyzed in a domain angular in 2θ from 20° to 55° in steps of 0.04°. The morphology of the layers was investigated using a scanning electron microscopy (SEM) Philips XL 30 SFEG microscope. It is an SEM with a spatial resolution of 1.2 nm, an OXFORD cryogenic stage, and an OXFORD cathodoluminescence.

### 2.4. Gas Sensor Measurement Calibration System

**Figure 2:**
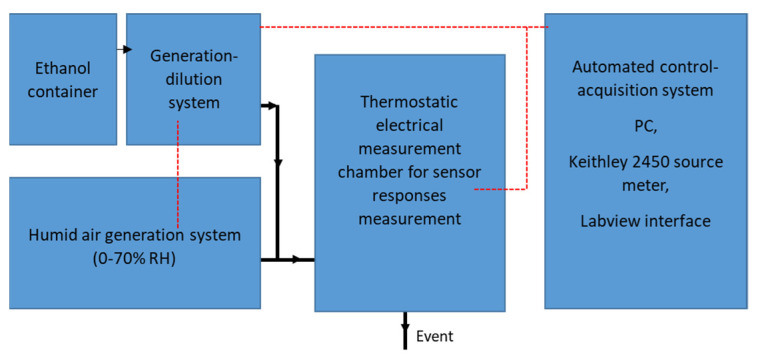
Schematic diagram of the test bench set up.

A small testing chamber with a total volume of 2.35 × 10^−3^ L was designed with the aim to approach transdermal detection conditions (more details are in [25]). The target gas (ethanol) was delivered to the testing chamber (Figure 2) via a gas generation–dilution system, making it possible to generate gases or gas mixtures in a controlled atmosphere. The dilution of the analytes was precisely controlled by the use of mass flow regulators, which makes it possible to generate an output mixture at very low concentrations (a few tens of ppb to a few tens of ppm). The output flow was adjustable within a range of 100 to 500 sccm (100 mL/min to 500 mL/min). The total flow rate was fixed at 100 sccm. The choice of this gas flow rate was related to the test chamber volume, in order to avoid turbulence. More details regarding the effect of the chamber volume on the gas flow rate are provided in ref. [25]. The gas sensing measurements were achieved by monitoring, in real time, the electrical resistance of the sensor via a Keithley 2450 source meter. A thermoregulated chamber was used in order to control the ambient temperature of the entire system (sensor + testing chamber). Regarding humidity system, the moist air was generated from a pressurized water container. The water was vaporized through a microporous membrane; the water vapor was injected into the carrier gas by means of a proportional valve. This proportional valve, assembled to a hygrometry probe placed at the humidifier outlet, makes it possible to keep the hygrometry of the mixture constant. Figure 2 depicts the schematic diagram of the test bench used in this work.

During measurements, the sensors were exposed to ethanol for 1 min and then the chamber was then purged with air until the recovery of the initial baseline resistance. The sensor response was determined as R = R_air_/R_gas_, where R_air_ is the sensor resistance in the air at a steady state and R_gas_ represents the sensor resistance after 1 min of the gas mixture exposure. The response time was defined as the time necessary for the sensor to reach 90% of the maximum value of its response upon the introduction of ethanol. Similarly, the recovery time was defined as the time required to recover to within 10% of the original baseline when the flow of ethanol gas is removed [27].

### 2.5. Transdermal Alcohol Sensing System Set Up

**Figure 3:**
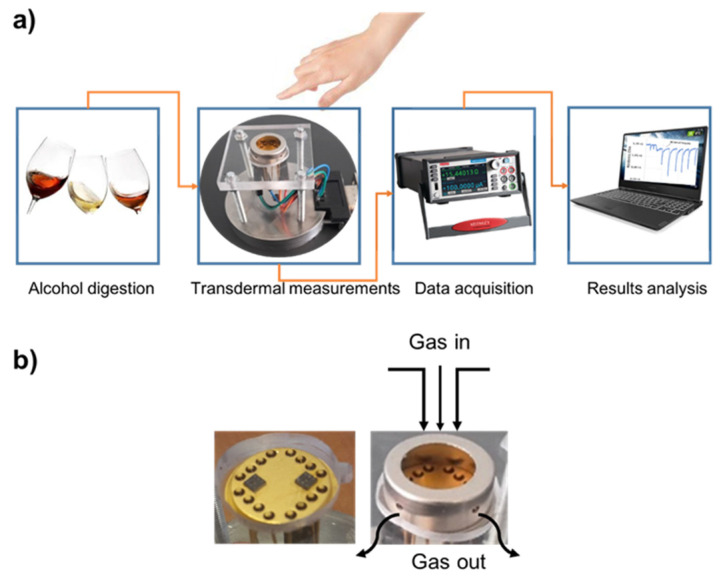
(**a**) Experimental set up of the transdermal alcohol detection and (**b**) the detection cell images.

The transdermal alcohol sensing system (Figure 3a) was built in our laboratory. It is composed of a homemade detection cell; a power supply to generate a sensor heating temperature; a multimeter Keithley 2450, to ensure the real time data acquisition of the sensor resistance; and a computer with LabVIEW interface to view the sensor responses. Figure 3b depicts the detection cell. It consists of TO-8 packaging, in which the MEMS-based microsensors are wire-bonded connectors, a homemade cover that has a small volume (2 × 10^−4^ L) with an open circle on the top (gas inlet), and four small holes in the wall (gas outlet). We have chosen this volume in order to collect a sufficient amount of ethanol vapor from the finger skin and to accelerate the sensor response.

### 2.6. DRAGER 6820 Certified Breathalyzer

To validate the transdermal alcohol measurements, the DRAGER 6820 certified breathalyzer was employed to measure the alcohol content in the exhaled air. This breathalyzer complies with the international regulations and has the EU approval for law enforcement (NF EN 15964) in other countries, such as Australia (AS 3547:1997) and the United States. It is equipped with stronger housing and a proven DRAGER sensor.

## 3. Results and Discussion

### 3.1. Thin Film Realization and Characterization

Tin dioxide is an n-type semiconductor with a wide band gap of 3.6 eV. It is one of the best known gas sensing materials that shows excellent chemical stability and a remarkable sensitivity to a wide spectrum of gases and vapors [28,29]. In this work, SnO_2_ films of 50 nm were successfully deposited via reactive radio frequency (RF) magnetron sputtering over the microsensor platforms. This deposition technique allows us the control of the ratio Ar/O_2_ in the plasma to modify the oxidation rate of the film. Based on our previous works [30], this ratio was set to 4/6, since it has shown the best gas sensing performances. After deposition at an ambient temperature, the films were transparent and very well adhered to the substrate. They were initially amorphous whereas, after the annealing treatment, they became crystalline, and their chemical structures were well stabilized. This treatment also ensures the improvement of the films’ sensing performances to a target gas (stability and sensitivity). In fact, we have fixed the annealing temperature to 500 °C and we have varied its duration to 2 h; 6 h; and 12 h, under dry air [23,31,32,33]. This study was conducted in order to optimize the sensing film fabrication parameters, which leads to a stable and very sensitive ethanol gas sensor.

**Figure 4:**
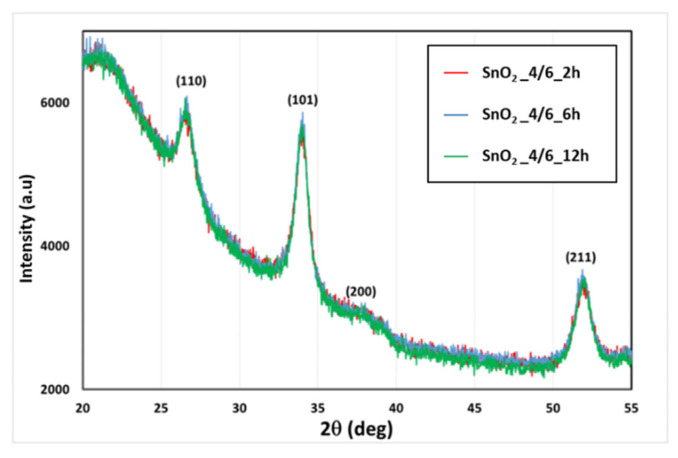
The XRD patterns recorded from the three annealed SnO_2_ samples.

Figure 4 depicts the X-ray diffraction patterns recorded from the three obtained samples. As we can observe, all the spectra showed the same peaks at 26.59° (110); 33.88° (101); 37.96° (200); and 51.79° (211). This was indicative of a tetragonal rutile phase of SnO_2_ with the mesh constants of a = b = 4.7370 A° and c = 3.18 A° (JCPDS N° 72–1147), with the preferred orientation at (101) and (110). Using the XRD analysis results, we noted that the structure of all the samples did not change with respect to the annealing time and that there were no significant changes in the recorded spectra.

**Figure 5:**
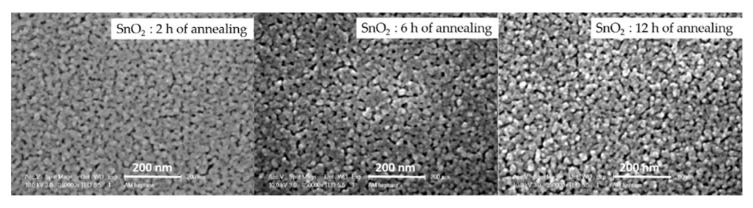
SEM images of the deposited samples at different annealing times.

The granular appearance and the porosity of the samples were investigated using scanning electron microscopy. Figure 5 displays the obtained results. By increasing the annealing duration, the size of the agglomerates (close packed crystallites) decreases and their boundaries sharpen and become more well defined. A similar microstructure behavior has been reported by Ryzhikov et al. [32] for SnO_2_. It is worth noting that better detection is generally obtained with nano-grains [34,35]. Moreover, the porosity rate seems to be enhanced by the annealing time, which increases the active surface and promotes surface-gas interactions. Therefore, the SnO_2_ sample with 12 h of annealing displays the best material characteristics that can lead to a very sensitive ethanol gas sensor. To confirm this hypothesis, we have exposed all three samples to different concentrations of ethanol at the same operating temperature of 300 °C.

**Figure 6:**
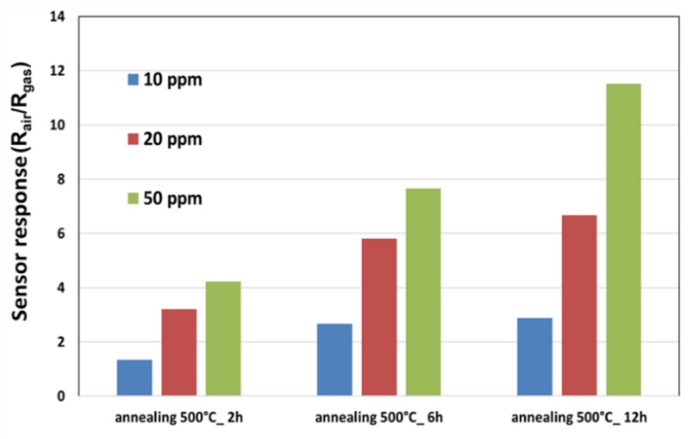
A comparison between the responses of sensors annealed for 2, 6, and 12 h and their exposure to different concentrations of ethanol.

Figure 6 shows the obtained the responses to 10 ppm, 30 ppm, and 50 ppm of ethanol in dry air. By increasing the annealing time, the ethanol sensitivity is highly increased. Consequently, all the fabricated sensors were annealed during 12 h under dry air.

### 3.2. Gas Sensing Results and Discussion

#### 3.2.1. Sensor Calibration Results

**Figure 7:**
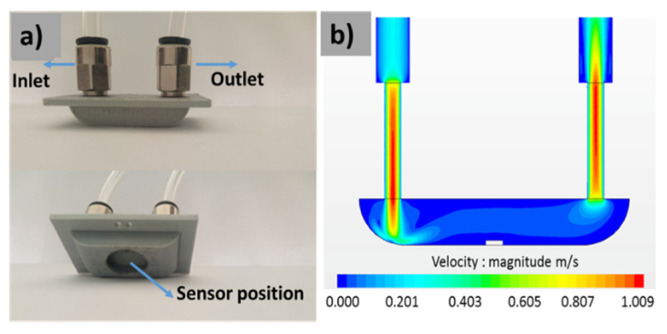
(**a**) Testing chamber photographs and (**b**) the finite element simulation of the gas flow inside the chamber.

As we have mentioned in the Section above, titled “Gas Sensor Measurement Calibration System”, the fabricated sensors need to be primarily studied in laboratory conditions before the transdermal measurements via the finger. This step helps us to improve our understanding of the sensor’s characteristics and to define its performances, including the concentration detection range, the optimal working temperature, and the influence of humidity on the sensor’s responses. Since the transdermal measurements will be carried out in a very small cell, as shown in Figure 3b, we have designed and fabricated a testing chamber (Figure 7) to remain near to the real measurement conditions, with the following technical characteristics: a small volume of 2.35 × 10^−3^ L; negligible dead volumes; homogeneous gas concentration in the test chamber; and a low gas flow rate around the sensor (more details are in [25]).

**Figure 8:**
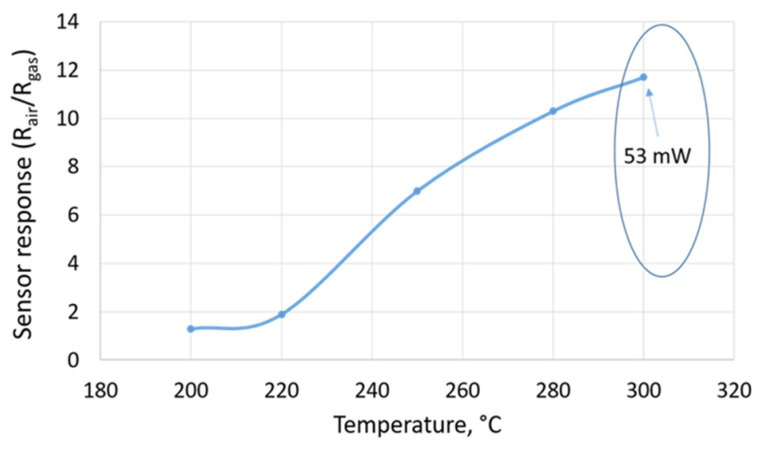
The response of the sensor as a function of a heater temperature.

The optimal working temperature is an important parameter to be determined for a gas sensing application. Indeed, it has a direct influence on the adsorption ability, catalytic activity, sensitivity, and response and recovery times [36]. Figure 8 displayed the behavior of the sensor as a function of a heater temperature in the presence of 50 ppm of ethanol. Overall, by increasing the temperature, the sensor response was increased, which means that the rate of adsorption was accelerated and the activation barrier was lowered. Furthermore, all the measurements were carried out at 300 °C, which corresponds to 53 mW, with an applied voltage of 2.1 V, in order to avoid the breaking of the membrane. This temperature value was considered to be the optimal working temperature for our sensors.

**Figure 9:**
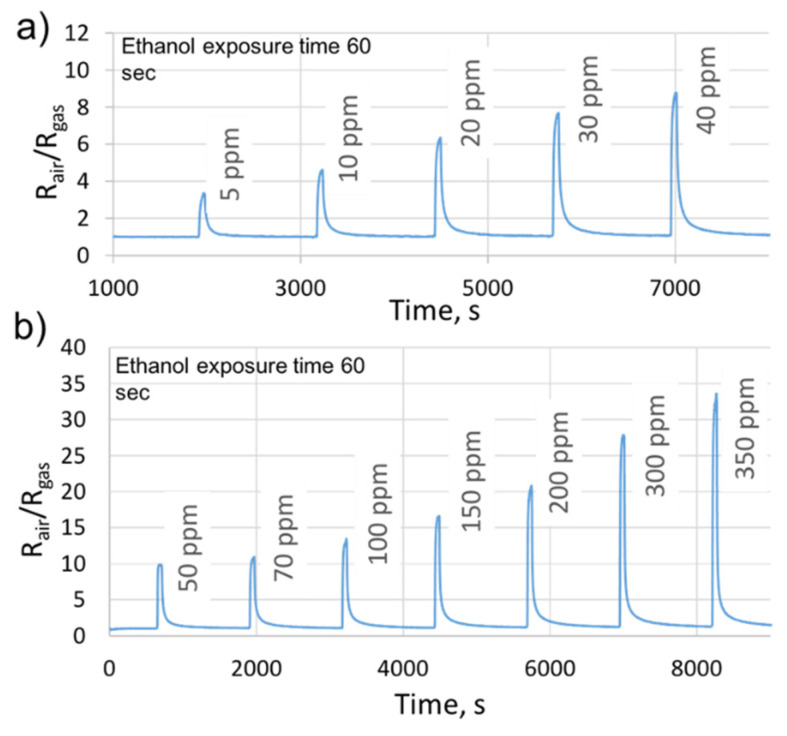
The gas sensor responses for (**a**) low and (**b**) high ethanol concentrations, under dry air, at the sensor’s optimal working temperature.

Moreover, we studied the sensitivity of the sensors to low and high concentrations of ethanol under dry air. The choice of concentration values was based on the different blood alcohol levels that can be encountered in the transdermal measurements. Figure 9 represents the obtained results. As we can observe, the sensor shows stable and very fast responses with good recovery times. For instance, at 200 ppm of ethanol, the response time was 23 s and the recovery time was 53 s. Moreover, the sensor behaves like an n-type semiconductor, i.e., decreasing the resistance when exposed to a reducing gas (ethanol). It was able to distinguish between all the studied concentrations. Additionally, at 5 ppm, the sensor displayed a clearly measurable response of 3.2, which means that the detection limit falls lower than the ppm level.

**Figure 10:**
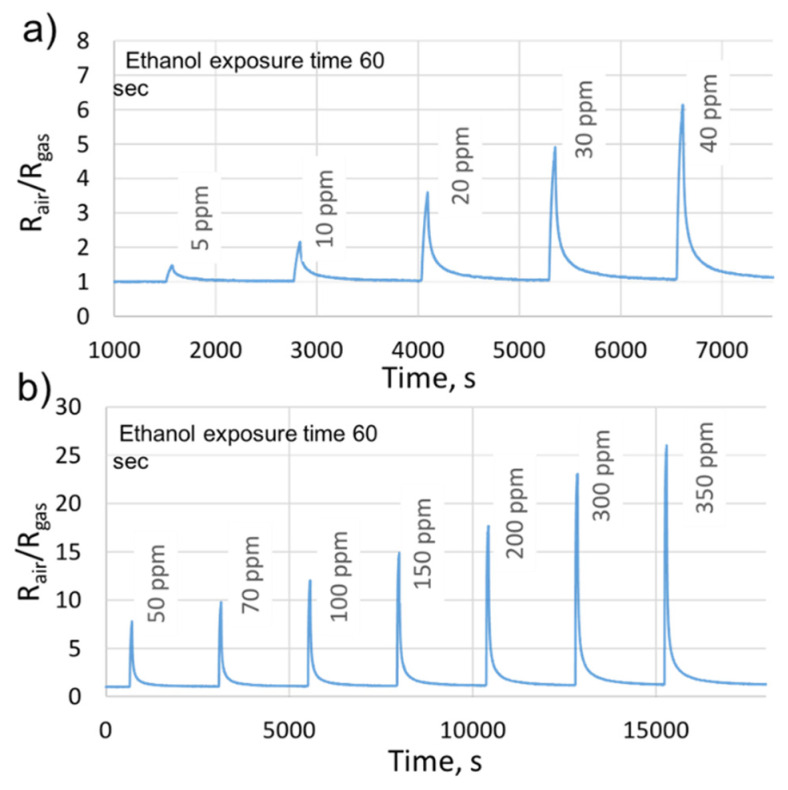
The gas sensor responses to (**a**) low and (**b**) high ethanol concentrations, under 70% of the RH, at the sensor’s optimal working temperature. Reprinted with permission from ref. [39]. Copyright 2019 IEEE.

Subsequently, we retained the same ethanol concentrations used in the previous study, and we have introduced 70% of the relative humidity (RH). In fact, the fabricated sensors are designed for the real application of ethanol transdermal detection, in which the relative humidity will be considered as an interfering gas. Thus, it is essential to study its impact on the sensors’ performances. Figure 10 indicates the sensor responses, as a function of time, to various ethanol concentrations in the presence of 70% of RH. We can notice that the sensor is functional even at this high level of relative humidity. The responses were stable with a slight decrease in their values compared to those obtained under dry air. Such a decrease is common for the majority of the metal oxides in the presence of humidity [27,37,38]. It is related to the adsorption competition between the target gas molecules and the hydroxyl group –OH, at the sensing film surface [27,38]. Moreover, for small concentrations (5–40 ppm), the time necessary to recover to the baseline resistance was equal to the one used in the presence of dry air (20 min). In contrast, it has been increased to 40 min, for concentrations ranging from 50 ppm to 350 ppm (70% of RH). Indeed, the desorption kinetic at 70% of RH is slow for high concentrations, and the sensing film requires more time to clean its surface from the target gas molecules and to recover to its initial baseline resistance.

**Figure 11:**
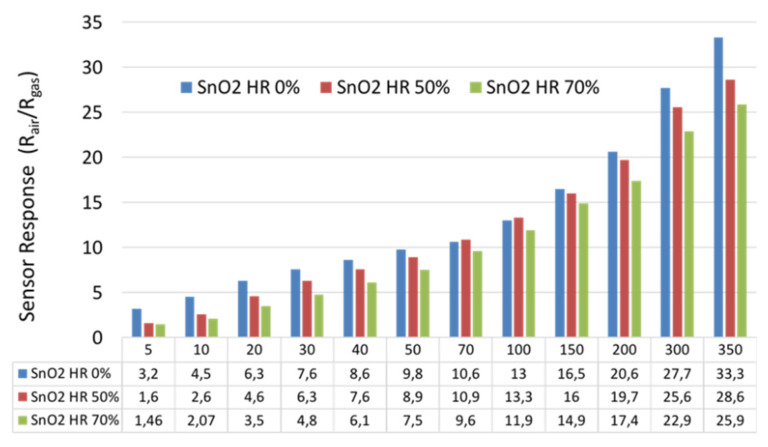
A comparison between the sensor responses to the presence of dry air at 50% of RH and 70% of RH.

Figure 11 represents a summary of the sensor responses to all the studied ethanol concentrations in dry and humid atmospheres. We can observe that the effect of humidity on the sensor’s performance was low. The SnO_2_ fabricated sensor shows a good immunity against high levels of relative humidity, which makes it an excellent candidate for our real application.

**Figure 12:**
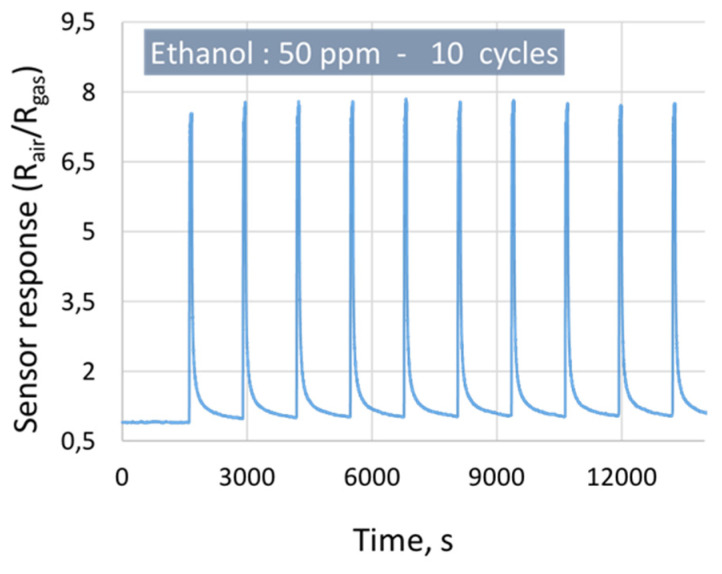
The gas sensor responses as a function of time, using 10 cycles of 50 ppm of ethanol at 70% of the RH.

Figure 12 shows the repeatability of the sensor response to 50 ppm of ethanol with 70% of RH, using 10 cycles of measurements at 300 °C.

The graph clearly shows that the sensor responses are reproducible with a small Standard Deviation (1) of 0.2 and Repeatability Error (2) of 2.5%.

(1) $$Sr=\sqrt{\frac{\sum {\left(X-X\right)}^{2}}{n}}$$

(2) $$Er\%=\left(\frac{Sr}{X}\right)\times 100$$

#### 3.2.2. Gas Sensing Mechanism

The gas sensing mechanism of SnO_2_ under ethanol is generally based on the adsorption and desorption of the gas molecules at the material surface, which lead to a change in its electrical resistance. When SnO_2_ is exposed to the air, oxygen molecules capture electrons from the conduction band of the materials and form ionized oxygen species, such as $O_{2}^{-}$, $O^{-}$, and $O^{2-}$. This reaction results in the formation of a depletion layer at the material surface, and a decrease in the concentration of the charge carriers leading to an increase in the material’s resistance. When the material is exposed to ethanol (a reducing gas), the adsorbed oxygen species interacts with the ethanol molecules, forming CO_2_ and H_2_O (Equations (3) and (4)), and the trapped electrons are released back towards the conduction band of the SnO_2_ material. This reaction leads to a thinner depletion layer and a decrease in the material’s resistance [40,41].

(3) $$3O_{2}^{-}+C_{2}H_{5}OH\;\;\;--\to \;3H_{2}O+2\;CO_{2}+3e^{-}$$

(4) $$3O_{2}^{2-}+C_{2}H_{5}OH\;\;\;--\to \;3H_{2}O+2\;CO_{2}+6e^{-}$$

#### 3.2.3. Real Time Transdermal Alcohol Detection Results

After the calibration of the sensor and the definition of its characteristics, a real time transdermal alcohol detection was performed using an adult volunteer, who was a research member of our group (no funding was applied).

**Figure 13:**
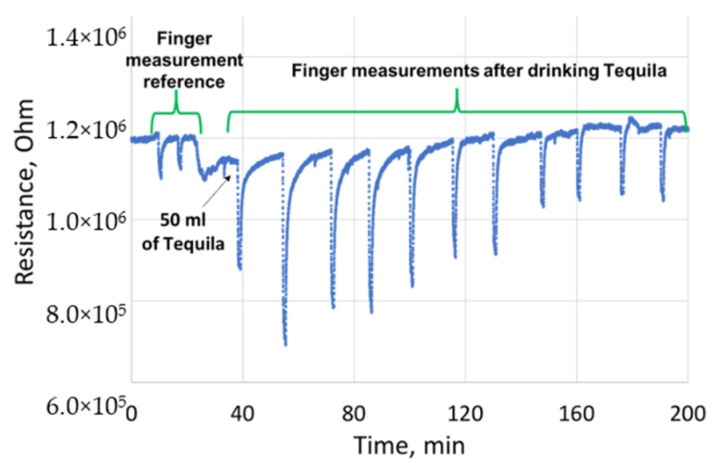
The gas sensor transdermal measurements, separated by 15 min, during 1 min of exposure. Reprinted with permission from ref. [39]. Copyright 2019 IEEE.

The measurement methods were organized as follows: first, the sensor reference was defined by placing the finger of the volunteer on top of the transdermal cell, several times. Next, the volunteer drank 50 mL of tequila, which corresponds to a target of approximatively 0.5 g/L of BAC. Subsequently, the volunteer started to record the measurements of the alcohol levels every 15 min with our SnO_2_ microsensor and with a certified DRAGER 6820 breathalyzer. This was later calibrated to directly give the blood alcohol concentration levels in g/L. It is worth noting that the sensor was continuously heated at its optimal working temperature. Additionally, the corresponding values of 5 ppm and 350 ppm of ethanol in the expired air in the breath analysis are 0.009 mg/L and 0.637 mg/L, respectively, while they correspond to 0.024 g/L and 1.639 g/L in the blood. Moreover, the distance between the finger and the sensor is kept constant and it corresponds to the height of the TO8 box, i.e., 0.3 cm. Figure 13 illustrates the sensor’s resistance change as a function of time, with different transdermal alcohol exposures. It appears a clear rise after drinking the tequila by 15 min, the sensor starts to detect ethanol emitted through the skin. Thirty minutes after drinking the tequila, the highest ethanol sensor response was observed, which corresponds to the maximum ethanol emission from the skin. Subsequently, a slow decrease in the sensor response was noticed over the remaining time of the experiment.

**Figure 14:**
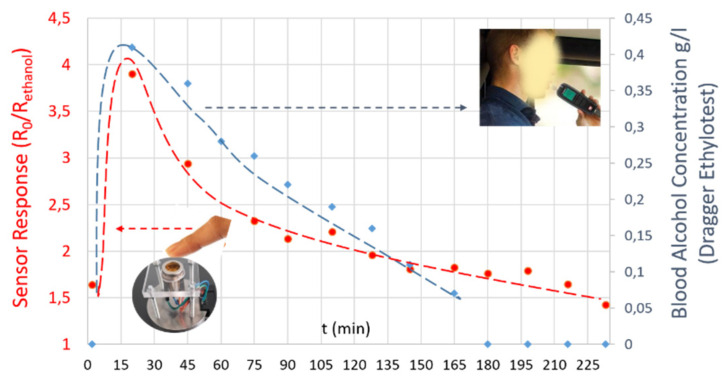
A comparative study between gas sensors responses obtained during the transdermal detection, and the blood alcohol concentration deduced from the DRAGER 6820 certified breathalyzer.

Figure 14 displays a comparative study between alcohol transdermal detection, using SnO_2_ gas sensors, and blood alcohol content, using a certified DRAGER 6820 breathalyzer. Following the ingestion of the tequila, the blood and transdermal tests show a rapid increase in blood alcohol levels and sensor responses, respectively. The time necessary to observe the alcohol level of 0.0 g/L in the blood was 3 h. In contrast, we noticed that the sensor was still detecting alcohol from the skin even after 4 h. Indeed, this slow kinetics of ethanol removal from the skin has already been mentioned in the literature [12]. Hence, there is a clear conformity between the obtained results, which validate our embedded transdermal detector device based on the SnO_2_ MEMS gas sensors.

## 4. Conclusions

In this work, we demonstrated that metal oxide gas sensors can be a good candidate to indirectly monitor the blood alcohol content using a human finger, via transdermal detection. Owing to their tiny size and their heater low power consumption levels, the MEMS-based microsensors have promoted the portability of the device and have overcome the drawbacks seen with the conventional techniques. We indicated that the annealing time has an important role in enhancing the sensing film performances. It has affected the porosity and the granular size of the SnO_2_ films. The gas sensing calibration results showed that the SnO_2_ films exhibited excellent ethanol gas sensing characteristics at very low heater power consumption (53 mW) levels; the sensors were very sensitive to small concentrations of ethanol with a fast detection and recovery time. Additionally, the detection limit fell in the range of the ppb level and the sensors were very resistant to high levels of relative humidity (50% and 70%). Finally, the transdermal alcohol detection results, obtained using a volunteer (an adult from our research group), demonstrated the feasibility of SnO_2_ gas sensors in the continuous monitoring of alcohol levels in the blood and evaporated through the skin. We noticed a strong accordance between the blood alcohol content and the transdermal alcohol content.

## Data Availability

Not Applicable.

## References

[1] Boffetta P., Hashibe M. (2006). Alcohol and cancer. Lancet Oncol..

[2] Cargiulo T. (2007). Understanding the health impact of alcohol dependence. Am. J. Health Pharm..

[3] Saitz R., Ghali W.A., Moskowitz M.A. (1997). The Impact of Alcohol-Related Diagnoses on Pneumonia Outcomes. Arch. Intern. Med..

[4] Jones L., Bates G., McCoy E., Bellis M.A. (2015). Relationship between alcohol-attributable disease and socioeconomic status, and the role of alcohol consumption in this relationship: A systematic review and meta-analysis. BMC Public Health.

[5] Singh S.P., Padhi P.K., Narayan J., Singh A., Pati G.K., Nath P., Parida P.K., Mishra S. (2016). Socioeconomic impact of alcohol in patients with alcoholic liver disease in eastern India. Indian J. Gastroenterol..

[6] Bendtsen P., Hultberg J., Carlsson M., Jones A.W. (1999). Monitoring ethanol exposure in a clinical setting by analysis of blood, breath, saliva, and urine. Alcohol. Clin. Exp. Res..

[7] Bates M.E., Brick J., White H.R. (1993). The correspondence between saliva and breath estimates of blood alcohol concentration: Advantages and limitations of the saliva method. J. Stud. Alcohol.

[8] Fosnight A.M., Moran B.L., Medvedev I.R. (2013). Chemical analysis of exhaled human breath using a terahertz spectroscopic approach. Appl. Phys. Lett..

[9] Bertholet N., Winter M.R., Cheng D.M., Samet J.H., Saitz R. (2014). How Accurate Are Blood (or Breath) Tests for Identifying Self-Reported Heavy Drinking Among People with Alcohol Dependence?. Alcohol Alcohol..

[10] Thungon P.D., Kakoti A., Ngashangva L., Goswami P. (2017). Advances in developing rapid, reliable and portable detection systems for alcohol. Biosens. Bioelectron..

[11] Hlastala M.P. (1998). The alcohol breath test—A review. J. Appl. Physiol..

[12] Lawson B., Aguir K., Fiorido T., Martini-Laithier V., Bouchakour R., Burtey S., Reynard-Carette C., Bendahan M. (2019). Skin alcohol perspiration measurements using MOX sensors. Sens. Actuators B Chem..

[13] Jones A.W. (1982). How Breathing Technique Can Influence the Results of Breath-Alcohol Analysis. Med. Sci. Law.

[14] Dougherty D.M., Charles N.E., Acheson A., John S., Furr R.M., Hill-Kapturczak N. (2012). Comparing the detection of transdermal and breath alcohol concentrations during periods of alcohol consumption ranging from moderate drinking to binge drinking. Exp. Clin. Psychopharmacol..

[15] Swift R.M., Martin C.S., Swette L., LaConti A., Kackley N. (1992). Studies on a wearable, electronic, transdermal alcohol sensor. Alcohol. Clin. Exp. Res..

[16] Kim J., Jeerapan I., Imani S., Cho T.N., Bandodkar A., Cinti S., Mercier P.P., Wang J. (2016). Noninvasive Alcohol Monitoring Using a Wearable Tattoo-Based Iontophoretic-Biosensing System. ACS Sens..

[17] Lansdorp B., Ramsay W., Hamid R., Strenk E. (2019). Wearable enzymatic alcohol biosensor. Sensors.

[18] Nyman E., Palmlöv A. (1936). The Elimination of Ethyl Alcohol in Sweat1. Skand. Arch. Physiol..

[19] Brown D.J. (1985). A method for determining the excretion of volatile substances through skin. Methods Find. Exp. Clin. Pharmacol..

[20] Anderson J.C., Hlastala M.P. (2006). The kinetics of transdermal ethanol exchange. J. Appl. Physiol..

[21] Alessi S.M., Barnett N.P., Petry N.M. (2017). Experiences with SCRAMx alcohol monitoring technology in 100 alcohol treatment outpatients. Drug Alcohol Depend..

[22] Simons J.S., Wills T.A., Emery N.N., Marks R.M. (2015). Quantifying alcohol consumption: Self-report, transdermal assessment, and prediction of dependence symptoms. Addict. Behav..

[23] Khan A.F., Mehmood M., Rana A.M., Bhatti M.T. (2009). Effect of annealing on electrical resistivity of rf-magnetron sputtered nanostructured SnO_2_ thin films. Appl. Surf. Sci..

[24] Ramarajan R., Kovendhan M., Thangaraju K., Joseph D.P., Babu R.R. (2019). Facile deposition and characterization of large area highly conducting and transparent Sb-doped SnO_2_ thin film. Appl. Surf. Sci..

[25] Annanouch F.-E., Bouchet G., Perrier P., Morati N., Reynard-Carette C., Aguir K., Martini-Laithier V., Bendahan M. (2019). Hydrodynamic evaluation of gas testing chamber: Simulation, experiment. Sens. Actuators B Chem..

[26] Annanouch F.-E., Bouchet G., Perrier P., Morati N., Reynard-Carette C., Aguir K., Bendahan M. (2018). How the Chamber Design Can Affect Gas Sensor Responses. Proceedings.

[27] Annanouch F.E., Haddi Z., Vallejos S., Umek P., Guttmann P., Bittencourt C., Llobet E. (2015). Aerosol-assisted CVD-grown WO_3_ nanoneedles decorated with copper oxide nanoparticles for the selective and humidity-resilient detection of H_2_S. ACS Appl. Mater. Interfaces.

[28] Vallejos S., Selina S., Annanouch F.E., Gràcia I., Llobet E., Blackman C. (2016). Aerosol assisted chemical vapour deposition of gas sensitive SnO_2_ and Au-functionalised SnO_2_ nanorods via a non-catalysed vapour solid (VS) mechanism. Sci. Rep..

[29] Vallejos S., Selina S., Annanouch F.E., Gràcia I., Llobet E., Blackman C. (2016). Micromachined Gas Sensors Based on Au-functionalized SnO_2_ Nanorods Directly Integrated without Catalyst Seeds via AA-CVD. Procedia Eng..

[30] Lawson B. (2018). Nouvelle Approche de suivi non Invasif de L’alcoolémie par Perspiration à l’aide de Multicapteurs MOX. Ph.D. Thesis.

[31] Mani G.K., Rayappan J.B.B. (2014). Impact of annealing duration on spray pyrolysis deposited nanostructured zinc oxide thin films. Superlattices Microstruct..

[32] Ryzhikov A.S., Vasiliev R.B., Rumyantseva M.N., Ryabova L.I., Dosovitsky G.A., Gilmutdinov A.M., Kozlovsky V.F., Gaskov A.M. (2002). Microstructure and electrophysical properties of SnO_2_, ZnO and In_2_O_3_ nanocrystalline films prepared by reactive magnetron sputtering. Mater. Sci. Eng. B.

[33] Beaurain A., Luxembourg D., Dufour C., Koncar V., Capoen B., Bouazaoui M. (2008). Effects of annealing temperature and heat-treatment duration on electrical properties of sol–gel derived indium-tin-oxide thin films. Thin Solid Films.

[34] Yamazoe N. (1991). New approaches for improving semiconductor gas sensors. Sens. Actuators B Chem..

[35] Katoch A., Sun G.-J., Choi S.-W., Byun J.-H., Kim S.S. (2013). Competitive influence of grain size and crystallinity on gas sensing performances of ZnO nanofibers. Sens. Actuators B Chem..

[36] Annanouch F.E., Gràcia I., Figueras E., Llobet E., Cané C., Vallejos S. (2015). Localized aerosol-assisted CVD of nanomaterials for the fabrication of monolithic gas sensor microarrays. Sens. Actuators B Chem..

[37] Alagh A., Annanouch F.E., Umek P., Bittencourt C., Sierra-Castillo A., Haye E., Colomer J.F., Llobet E. (2021). CVD growth of self-assembled 2D and 1D WS_2_ nanomaterials for the ultrasensitive detection of NO_2_. Sens. Actuators B Chem..

[38] Merdrignac-Conanec O., Bernicot Y., Guyader J. (2000). Humidity effect on baseline conductance and H2S sensitivity of cadmium germanium oxynitride thick film gas sensors. Sens. Actuators B Chem..

[39] Annanouch F.E., Aguir K., Martini-Laithier V., Fiorido T., Bendahan M. SnO_2_ Sensors for a Portable Transdermal Alcohol Detector Via Finger. Proceedings of the IEEE Sensors.

[40] Li Z., Yi J. (2017). Enhanced ethanol sensing of Ni-doped SnO_2_ hollow spheres synthesized by a one-pot hydrothermal method. Sens. Actuators B Chem..

[41] Zhang B., Fu W., Li H., Fu X., Wang Y., Bala H., Wang X., Sun G., Cao J., Zhang Z. (2016). Synthesis and characterization of hierarchical porous SnO_2_ for enhancing ethanol sensing properties. Appl. Surf. Sci..

